# Notes and Notices

**Published:** 1897-10

**Authors:** 


### NOTES AND NOTICES.

STAMFORD HALL.—Of the many Sanitariums for the treatment of Mental and Nervous Disease, that of Dr. Givens at Stamford, Conn., possesses unexceptional advantages.

Built on the cottage plan, nervous and mental patients can be more satisfactorily cared for than the old plan of many under one roof; and the Doctor is fortunate in having a location so accessible to New York and in such close proximity to the seashore.

